# Satisfaction of family physician team members in the context of contract system: A cross-sectional survey from Shandong Province, China

**DOI:** 10.3389/fpubh.2022.1000299

**Published:** 2022-11-25

**Authors:** Weiqin Cai, Yuanze Du, Qianqian Gao, Runguo Gao, Hongqing An, Wenwen Liu, Fang Han, Qi Jing, Chunping Wang

**Affiliations:** ^1^School of Management, Weifang Medical University, Weifang, China; ^2^School of Public Health, Weifang Medical University, Weifang, China; ^3^Department of Organization and Human Resource, Affiliated Hospital of Weifang Medical University, Weifang, China; ^4^Department of Pathology, Affiliated Hospital of Weifang Medical University, Weifang, China

**Keywords:** family physician teams, job satisfaction, contract system, cross-sectional survey, China family physician team members

## Abstract

**Background:**

Family physicians play a key role in responding to the growing demand for primary healthcare due to aging. The work attitude of family physician team members (FPTMs) impacts their work efficiency and quality. Knowing how satisfied they are with their jobs can help identify potential directions and entry points for incentives. The purpose of this study is to analyze the job satisfaction status and influencing factors of grassroots health service personnel after the implementation of the family physician contract system in China.

**Methods:**

The study conducted a cross-sectional survey with 570 FPTMs in three prefecture-level cities in the Shandong Province. Satisfaction was measured using 30 items across seven dimensions. Responses were recorded on a 5-point Likert scale. Descriptive statistical analysis was used to analyze the general information and satisfaction of FPTMs. Multiple linear regression analysis was used to analyze the factors influencing job satisfaction.

**Results:**

The overall job satisfaction among FPTMs was not high. Among the seven dimensions, interpersonal relationships had the highest satisfaction (4.10 ± 0.78), while workload had the lowest satisfaction (3.08 ± 0.56). The satisfaction levels of the three sample regions were different, and the results were opposite to their regional economic development levels. The results of the multifactor analysis showed that gender, income level, educational background, working years, daily working hours, number of training sessions per year and the proportion of performance pay had significant impact on overall job satisfaction.

**Conclusions:**

The development of a family physician contract system has increased the workload of FPTMs. In addition to the implementation of the new policy, attention should be paid to the workload and working attitude of family physicians. The fundamental measures should focus on attracting more personnel to work at the grassroots level by accelerating education and training to solve the problem of insufficient health personnel at the grassroots level. Simultaneously, attention should be paid to the improvement of the medical staff's salary level and the need for self-promotion, such as training.

## Introduction

As the aging process accelerates, demand for primary healthcare services continues to increase (1). Family physicians and general practitioners have always been the main providers of primary healthcare and the fundamental building blocks of the healthcare system, especially in response to the increasing demand for primary healthcare due to aging (2). Currently, many countries and regions have established an optimal family physician system (3–5). The family physician system is a model that provides continuous, safe, effective, and appropriate comprehensive medical and health services and health management for residents in the community by means of contract services with general practitioners at the core (6). By signing a contract with residents and implementing the first consultation system, family physicians have become gatekeepers of residents' health and have played an important role in standardizing the utilization of health resources and reducing residents' medical expenses (7, 8).

The work attitudes of family medical team members have an impact on their work efficiency and quality. Those who are satisfied with their work can maintain high enthusiasm, put more energy into their work, and are willing to continue working on their own posts to complete medical service work (9, 10). Investigating the satisfaction of family physicians is conducive to mastering their job satisfaction status and determining the potential direction and entry point of incentive measures (11). Recent studies on the satisfaction of family physicians and general practitioners provide a good reference for conducting research (12). For example, one study examined the job satisfaction of 7,379 general practitioners in 34 countries and confirmed a wide variation in job satisfaction between countries (13). A study that analyzed mental health and job satisfaction among general practitioners in Denmark showed a high prevalence of poor mental health and low job satisfaction, especially among mid-career general practitioners and male general practitioners (14). A cross-country study examined the working conditions and career satisfaction of female family physicians, who play an increasingly important role in family medicine. Although they have higher cumulative satisfaction, they are less satisfied in terms of pay, personal time, and administrative tasks (15). In recent years, many studies have focused on doctors' job satisfaction or professional identity, but few have focused on the satisfaction of family physicians or general practitioners (16). Moreover, most studies have focused on the satisfaction of family physicians or general practitioners alone, and there is a lack of satisfaction surveys regarding all family physicians.

China's family physician system started relatively late. In 2016, the Guiding Opinions on Promoting Contracted Family Physician Services were predominantly aimed at speeding up contracted family physician services in combination with the comprehensive reform of community-level medical and health institutions, and the construction of the general practitioner system (17). Since then, family physician contract services have been promoted in all parts of China. On the one hand, the implementation of this policy has enabled the contracted residents to receive more adequate primary health care services (18). Simultaneously, it plays an important role in establishing a reasonable and orderly medical order. At present, the promotion of the signing policy in various regions is mainly carried out by family physician teams. The family physician team is mainly composed of family physicians, nurses, and public health practitioners (assistants). Family physicians include general practitioners, practicing (assistant) doctors in township health centers and community health service institutions, rural doctors who have obtained the qualification of practicing (assistant) doctors, and retired clinicians in public hospitals. The funds for contracted services include social medical insurance funds, financial funds for national basic public health service projects, and contracted residents' fees. According to the policy, no < 70% of the contracted service fee is used for the salary distribution of the family physician contracted service personnel, and the contracted service fee will be paid after the task performance assessment is qualified.

The signing system is a new policy, which not only brings performance income to grass-roots medical personnel, but also brings new workload. With the development of contracted services, a shortage of family physicians in China has also been exposed. Statistics show that China is still far from its goal of having five qualified general practitioners for every 10,000 urban and rural residents by 2030 (19). In addition, family physician team members (FPTMs) need to complete not only household services specified in the contract, but also a lot of daily clinical work, as well as many public health services, including residents' establishment of health records, provision of personalized health guidance services, outpatient appointment services and two-way referral services (20). As the provider of contracted services, the family physician service team plays the role of “gatekeeper” of the residents' health, and its teamwork efficiency and service quality are key to the smooth implementation of contracted services. It is necessary to pay attention to the job satisfaction of family physician teams in a timely manner when the number of human resources is small and the task load is heavy (21).

This study conducts a survey with FPTMs to analyze the current situation and influencing factors of job satisfaction of FPTMs in China under the condition of more work tasks than that before China began to implement the new policy of signing contracts. Additionally, we explore the differences in job satisfaction of FPTMs across different dimensions, providing a reference for improving the overall efficiency and service quality of FPTMs, and encouraging institutions to implement incentive measures.

## Methods

### Study design and data sources

Multistage stratified random sampling was used within this study. Jinan City, Weifang City, and Heze City of Shandong Province were selected based on their economic development levels. Counties or districts were randomly selected from each district. Three community service centers and one township central health center were selected from each county (district). A specific sampling plan, combined with the actual local situation, adopted the local community service agency manager's opinion and selected the appropriate agency. All members of the family physician team in the sample institutions were surveyed. Members include general practitioners, nurses, public health personnel, pharmacists, and psychological counselors. A total of 570 members of the family physician team participated in the survey. Among them, 179 were from Jinan, 148 were from Weifang, and 243 were from Heze.

### Measurement

The questionnaire was adapted from the short Minnesota Satisfaction Questionnaire developed by Weiss, Dawis, England, and Lofquist (22), which is a well-known instrument over the time and quite reliable. We used the Chinese version of the questionnaire. It includes demographic information and questions related to job satisfaction. The job satisfaction measurement includes seven dimensions: workload, training and career development, remuneration, working conditions and environment, interpersonal relationships, management systems, and social achievement. There were a total of 30 items. This is similar to the measurements used in other studies (23). Responses were provided on a 5-point Likert scale, with 1 being “least satisfied” and 5 being “most satisfied.” The Cronbach's alpha coefficient of the questionnaire was 0.902, indicating that the questionnaire had good reliability.

After the questionnaire was completed, we conducted a pre-survey in Weicheng District, Weifang City. In view of the problems in the pre-survey process, we modified the questionnaire. The formal investigation was conducted between January and February 2018. The investigation was led and assisted by the local health administrator, and the investigation team found and completed a survey of all family physician team members. During the survey, members of the family physician team completed a self-administered questionnaire, which was then examined one by one by an investigator. If the investigator found any vacant items, the questionnaire was sent to the respondents and corrected.

### Statistical analyses

Epidata 3.1 software was used to establish the database and input the questionnaire. Statistical analysis was performed using IBM SPSS Statistics 24 software. General data and satisfaction with FPTMs were analyzed using descriptive statistical analysis. In the absence of data for a variable, the effective percentage was calculated. The differences in satisfaction with FPTMs in general and among different regions were demonstrated using radar maps. Multiple linear regression was used to analyze the factors influencing job satisfaction, including cumulative satisfaction and the factors influencing each dimension of satisfaction. The significance level of the *P*-value was set to be 0.05.

## Results

### General information characteristics of FPTMs

As shown in Table 1 570 FPTMs were investigated in this study, including 242 males (42.4%) and 326 females (57.1%). Most of the FPTMs were aged 45 years and below, accounting for 84.8%. The income of most FPTMs was concentrated at 2001–4000 yuan, accounting for 77.4%. Junior college is the main degree of education. Junior professional title accounted for the highest proportion of 38.0%. The number of people who had worked for < 10 years was the largest. The working hours per day were mainly 6–8 h, but the number and proportion of people who work more than 8 h requires attention. Most FPTMs participated in training 1–3 times a year. Performance-based pay between 26–50% accounted for the majority (52.1%). The distribution of FPTMs with different basic information characteristics was statistically significant in the different regions (*P* < 0.05) (Table 1).

**Table 1:** Basic characteristics information of FPTMs investigated.

**Variable**	**Total ** **(*N* = 570)**	**Jinan** ** (*N* = 179)**	**Weifang** ** (*N* = 148)**	**Heze** ** (*N* = 243)**	**χ^2^**	***P*–value**
	***n (%)***	***n (%)***	***n (%)***	***n (%)***		
Gender^*^						
Male	242 (42.5)	97 (40.1)	39 (16.1)	106 (43.8)	26.001	< 0.001
Female	326 (57.2)	82 (25.2)	109 (33.4)	135 (41.4)		
Age (years)^*^						
≤ 35	224 (39.3)	43 (19.2)	63 (28.1)	118 (52.7)	45.020	< 0.001
36–45	260 (45.6)	99 (38.1)	77 (29.6)	84 (32.3)		
46–55	67 (11.8)	33 (49.3)	8 (11.9)	26 (38.8)		
≥56	11 (1.9)	3 (27.3)	0 (0.0)	8 (72.7)		
Income level (yuan)^*^						
≤ 2000	31 (5.4)	14 (45.2)	6 (19.4)	11 (35.4)	96.560	< 0.001
2001–3000	287 (50.4)	83 (28.9)	53 (18.5)	151 (52.6)		
3001–4000	155 (27.2)	60 (38.7)	31 (20.0)	64 (41.3)		
>4000	89 (15.6)	22 (24.7)	58 (65.2)	9 (10.1)		
Education^*^						
Junior high school and below	12 (2.1)	1 (8.3)	1 (8.3)	10 (83.4)	236.524	< 0.001
High school	159 (27.9)	8 (5.0)	22 (13.8)	129 (81.2)		
Junior college	214 (37.5)	53 (24.8)	69 (32.2)	92 (43)		
Undergraduate or above	183 (32.1)	117 (63.9)	56 (30.6)	10 (5.5)		
Professional title^*^						
No/other	155 (27.2)	12 (7.7)	41 (26.5)	102 (65.8)	98.829	< 0.001
Junior title	217 (38.1)	64 (29.5)	59 (27.2)	94 (43.3)		
Intermediate title	182 (31.9)	93 (51.1)	47 (25.8)	42 (23.1)		
Deputy senior title or above	13 (2.3)	10 (76.9)	1 (7.7)	2 (15.4)		
Working years^*^						
< 10	237 (41.6)	31 (13.1)	53 (22.4)	153 (64.5)		
11–20	178 (31.2)	75 (42.1)	50 (28.1)	53 (29.8)	102.861	< 0.001
21–30	112 (19.6)	53 (47.3)	38 (33.9)	21 (18.8)		
>30	19 (3.3)	11 (57.9)	0 (0.0)	8 (42.1)		
Daily working hours^*^						
6–8	378 (66.3)	100 (26.4)	119 (31.5)	159 (42.1)	23.343	< 0.001
9–11	162 (28.4)	68 (42.0)	25 (15.4)	69 (42.6)		
≥11	23 (4.0)	11 (47.9)	3 (13.0)	9 (39.1)		
Average number of residents served per month^*^						
≤ 300	282 (49.5)	102 (36.2)	77 (27.3)	103 (36.5)	19.131	0.004
301–600	188 (33.0)	57 (30.3)	47 (25.0)	84 (44.7)		
601–900	45 (7.9)	7 (15.6)	7 (15.6)	31 (68.8)		
>900	54 (9.5)	13 (24.1)	16 (29.6)	25 (46.3)		
Average number of patients referred per month^*^						
≤ 30	455 (79.8)	154 (33.9)	123 (27)	178 (39.1)	13.348	0.010
31–60	34 (6.0)	8 (23.5)	8 (23.5)	18 (53.0)		
>60	79 (13.9)	16 (20.3)	16 (20.3)	47 (59.4)		
Average number of door–to–door services per month^*^						
≤ 100	307 (53.9)	120 (39.1)	99 (32.2)	88 (28.7)	71.859	< 0.001
101–200	148 (26.0)	45 (30.4)	32 (21.6)	71 (48.0)		
>200	114 (20.0)	14 (12.3)	16 (14.0)	84 (73.7)		
Number of training sessions per year^*^						
No	77 (13.5)	41 (53.2)	30 (39.0)	6 (7.8)	129.050	< 0.001
1–3	251 (44.0)	112 (44.6)	64 (25.5)	75 (29.9)		
≥4	241 (42.3)	26 (10.8)	54 (22.4)	161 (66.8)		
Proportion of performance salary^*^						
≤ 25	119 (20.9)	14 (11.8)	69 (58.0)	36 (30.2)	157.207	< 0.001
26–50	297 (52.1)	134 (45.1)	61 (20.5)	102 (34.4)		
51–75	87 (15.3)	26 (29.9)	15 (17.2)	46 (52.9)		
>75	52 (9.1)	2 (3.8)	0 (0.0)	50 (96.2)		

* Calculated the effective percentage if missing data exists.

### Job satisfaction scores of FPTMs

As shown in Table 2, the cumulative satisfaction score of the FPTMs was 3.54 ± 0.63. Among the seven dimensions, interpersonal relationships scored the highest, with an average score of 4.10 ± 0.78, and was the only dimension that exceeded 4 points. The satisfaction of workload dimension was the lowest, with an average score of 3.08 ± 0.56. The other five dimensions remained between 3 and 4. In terms of specific items, the highest satisfaction degree was “Q7: Usefulness of training” in the dimension of training and career development, and “Q20: relationship with colleagues” in the dimension of interpersonal relationships, scoring 4.18 ± 0.87 and 4.18 ± 0.78, respectively, followed by “Q21: relationship with patients/residents,” scoring 4.13 ± 0.79. The problems with the lowest satisfaction were “work pressure” and “work difficulty” in the workload dimension, scoring 2.32 ± 0.78 and 2.54 ± 0.81 respectively.

**Table 2:** Job satisfaction scores of FPTMs.

**Dimension/item**	**Total** ** (*N* = 570)**	**Jinan** ** (*N* = 179)**	**Weifang (*N* = 148)**	**Heze** **(*N* = 243)**
	**(M ±SD)**	**(M ±SD)**	**(M ±SD)**	**(M ±SD)**
Workload	3.08 ± 0.56	2.94 ± 0.60	3.09 ± 0.42	3.18 ± 0.59
Q1: Task quantity	3.49 ± 0.84	3.25 ± 0.78	3.50 ± 0.81	3.66 ± 0.86
Q2: Work and rest Schedule	3.27 ± 0.96	2.97 ± 1.04	3.12 ± 0.78	3.59 ± 0.92
Q3: Working pressure	2.32 ± 0.78	2.34 ± 0.81	2.34 ± 0.77	2.29 ± 0.77
Q4:Workflow	3.84 ± 0.95	3.53 ± 1.00	3.87 ± 0.85	4.04 ± 0.90
Q5:Work difficulty	2.54 ± 0.81	2.62 ± 0.79	2.62 ± 0.70	2.44 ± 0.88
Training and career development	3.71 ± 0.79	3.38 ± 0.78	3.71 ± 0.79	3.96 ± 0.72
Q6: Equality of training opportunities	3.75 ± 0.98	3.39 ± 1.00	3.68 ± 1.01	4.07 ± 0.83
Q7: Usefulness of training	4.18 ± 0.85	3.97 ± 0.92	4.24 ± 0.86	4.29 ± 0.75
Q8: Training cost bearing method	3.88 ± 1.04	3.53 ± 1.11	3.97 ± 1.05	4.08 ± 0.92
Q9: Career development opportunities	3.51 ± 1.03	3.16 ± 1.07	3.48 ± 1.06	3.80 ± 0.88
Q10: Ways of professional title promotion	3.37 ± 1.05	2.98 ± 1.13	3.32 ± 0.96	3.68 ± 0.93
Remuneration	3.33 ± 0.87	3.00 ± 0.97	3.46 ± 0.74	3.50 ± 0.80
Q11: Salary income level	3.26 ± 0.97	2.95 ± 0.98	3.26 ± 0.96	3.49 ± 0.90
Q12: Financial subsidy level of essential drugs policy	3.43 ± 1.01	2.96 ± 1.12	3.54 ± 0.82	3.71 ± 0.91
Q13: Financial subsidy level of basic public health services	3.43 ± 1.02	3.03 ± 1.10	3.47 ± 0.90	3.71 ± 0.92
Q14: Welfare benefits of the institution	3.28 ± 1.07	2.97 ± 1.12	3.33 ± 1.01	3.48 ± 1.03
Q15: Pension security	3.32 ± 1.06	3.08 ± 1.07	3.73 ± 0.84	3.25 ± 1.10
Working conditions and environment	3.70 ± 0.87	3.39 ± 0.87	3.73 ± 0.77	3.90 ± 0.86
Q16: Working conditions of the institution	3.78 ± 0.93	3.4 ± 0.95	3.85 ± 0.91	4.00 ± 0.84
Q17: Living environment around the institution	3.7 ± 0.94	3.48 ± 0.94	3.56 ± 0.99	3.95 ± 0.85
Q18: Cultural construction of the institution	3.69 ± 0.98	3.34 ± 1.03	3.85 ± 0.91	3.98 ± 0.92
Interpersonal relationship	4.10 ± 0.78	3.88 ± 0.71	4.16 ± 0.79	4.23 ± 0.78
Q19: Relationship with superiors and subordinates	4.03 ± 0.83	3.76 ± 0.82	4.10 ± 0.88	4.20 ± 0.76
Q20: Relationship with colleagues	4.18 ± 0.78	3.94 ± 0.77	4.24 ± 0.85	4.32 ± 0.70
Q21: Relationship with patients/residents	4.13 ± 0.79	3.93 ± 0.75	4.14 ± 0.87	4.27 ± 0.75
Management system	3.73 ± 0.88	3.35 ± 0.91	3.85 ± 0.76	3.95 ± 0.84
Q22: Management system of the institution	3.76 ± 0.96	3.45 ± 1.02	3.76 ± 0.93	4.00 ± 0.86
Q23:Reward and punishment system of the institution	3.68 ± 0.97	3.23 ± 1.02	3.84 ± 0.85	3.92 ± 0.88
Q24: Performance salary assessment standard	3.67 ± 0.97	3.22 ± 1.03	3.79 ± 0.87	3.92 ± 0.86
Q25: Implementation of superior policies in the institution	3.8 ± 0.91	3.45 ± 1.00	3.85 ± 0.84	4.03 ± 0.79
Q26: Timely response from relevant departments	3.83 ± 0.96	3.41 ± 1.06	3.99 ± 0.88	4.04 ± 0.82
Social achievement	3.38 ± 0.75	3.14 ± 0.73	3.43 ± 0.72	3.53 ± 0.73
Q27: Sense of achievement in work	3.4 ± 0.91	3.13 ± 0.91	3.42 ± 0.90	3.60 ± 0.86
Q28: The future of family physicians	3.38 ± 0.96	3.11 ± 1.02	3.44 ± 0.99	3.55 ± 0.85
Q29: Local people's respect for you	3.63 ± 0.78	3.37 ± 0.80	3.78 ± 0.75	3.73 ± 0.73
Q30: Social status of family physicians	3.12 ± 0.82	2.94 ± 0.81	3.08 ± 0.79	3.28 ± 0.82
Cumulative satisfaction^*^	3.54 ± 0.63	3.26 ± 0.66	3.60 ± 0.54	3.71 ± 0.59

* Cumulative satisfaction score refers to average satisfaction scores of all dimensions.

**Figure 1:**
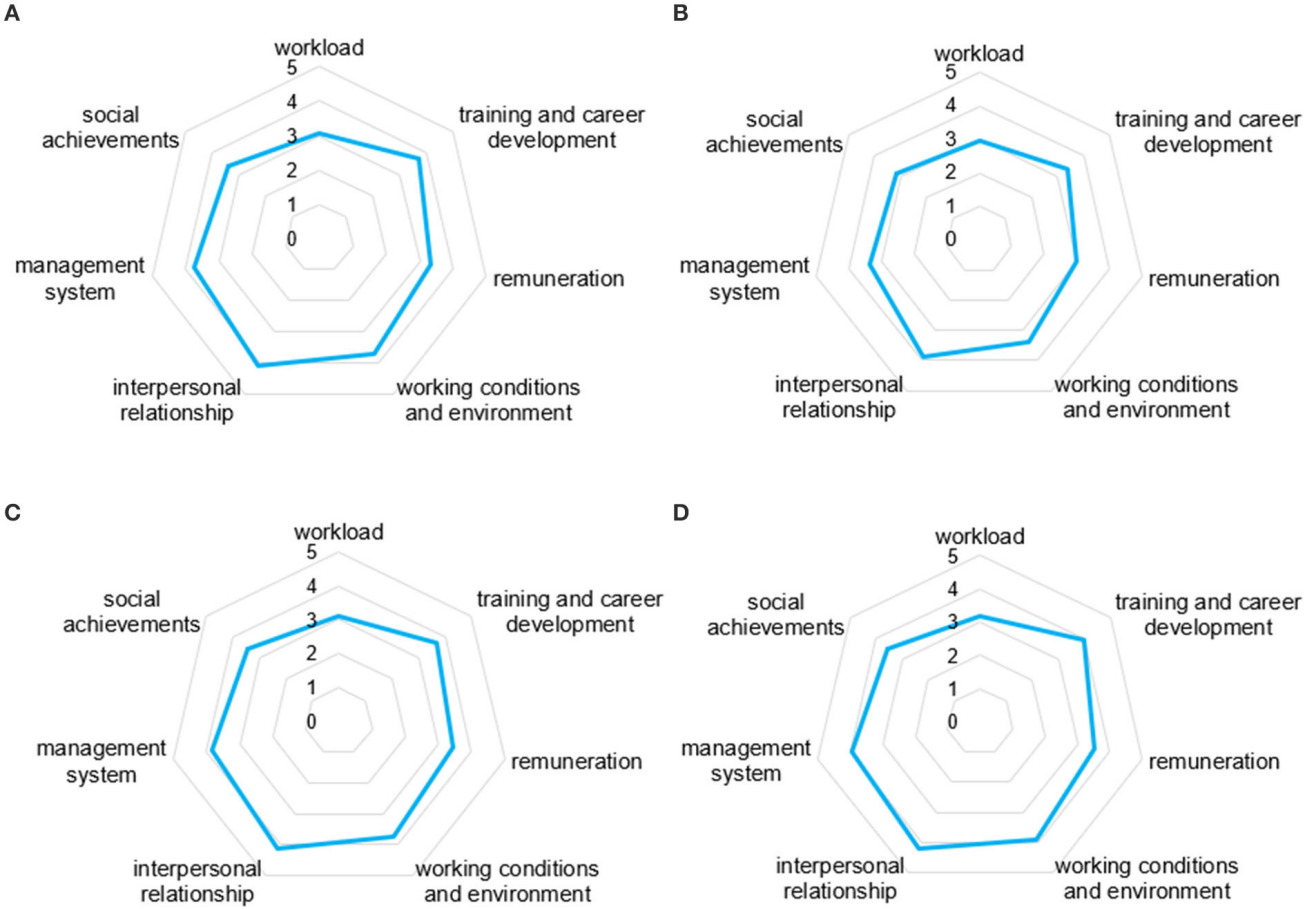
Radar chart of job satisfaction results of FPTMs. **(A)** In general and three regions, **(B)** Jinan, **(C)** Weifang, and **(D)** Heze.

The radar chart in Figure 1 shows the satisfaction levels in general and for each of the three regions. As shown in Figure 1, there are differences in satisfaction results in the sample regions, and the satisfaction results of FPTMs in different regions were the opposite to the regional economic development level. Jinan, with the best economic level, had the lowest degree of satisfaction in all dimensions, especially in the workload dimension, where the average degree of satisfaction was 2.94, which was lower than 3.0. Heze, which had the lowest economic level, had the highest satisfaction in all dimensions, especially in interpersonal relationships, with an average of 4.23. Weifang, which has a medium economic level, has a medium level of satisfaction in all dimensions among the three regions, which tends to be consistent with the overall level.

### Multifactor analysis of job satisfaction

Table 3 shows the influence factors of the seven dimensions and cumulative satisfaction. The results demonstrated that the number of training sessions affected the seven dimensions and cumulative satisfaction. There was a significant difference in gender and a positive correlation in six of the dimensions, except the workload dimension. Age was negatively correlated with workload (−0.080, 95%CI:−0.141-−0.019). Seven factors affecting cumulative satisfaction were identified: gender, income level, education, working years, daily working hours, number of training sessions per year, and the proportion of performance pay. Among them, females had higher satisfaction levels than males (0.182, 95%CI: 0.084–0.280). Income level (0.092, 95%CI: 0.029–0.156), number of training sessions (0.219, 95%CI: 0.145–0.292), and proportion of performance pay (0.058, 95%CI: 0.001–0.116) were positively correlated with cumulative satisfaction, indicating that the higher the income level, the more training sessions, and the higher the proportion of performance pay, the higher the job satisfaction. Education (−0.091, 95% CI: −0.155-−0.027), working years (0.116, 95% CI: 0.175–0.056), and daily work time (0.180, 95% CI:−0.270-−0.091) were negatively correlated with cumulative satisfaction. This indicates that higher education, longer working years, and longer working hours are associated with lower job satisfaction. The tolerance of all influencing factors in the equation was > 0.1, and the variance inflation factor was < 5, indicating that the established regression equation did not have a significant collinearity problem.

**Table 3:** Multi–factor analysis of FPTMs' job satisfaction.

	**Workload**		**Training and career development**		**Remuneration**	
	**B(95%CI)**	AB^*^(95%CI)	**B(95%CI)**	AB^*^(95%CI)	**B(95%CI)**	AB^*^(95%CI)
Gender	0.129 (0.036–0.222)		0.194 (0.063–0.326)	0.124 (0.002–0.245)	0.389 (0.247–0.530)	0.301 (0.162–0.440)
Age	−0.095 (−0.157–−0.032)	−0.080 (−0.141–−0.019)	−0.134 (−0.223–0.045)		−0.082 (−0.179–0.015)	
Income level	−0.026 (−0.083–0.031)		−0.009 (−0.089–0.071)		0.071 (−0.017–0.158)	0.149 (0.060–0.239)
Education	−0.105 (−0.160–−0.050)	−0.066 (−0.123–−0.010)	−0.206 (−0.283–−0.129)	−0.082 (−0.159–−0.006)	−0.177 (−0.262–−0.092)	−0.109 (−0.199–−0.018)
Working years	−0.085 (−0.138–−0.031)		−0.214 (−0.289–−0.139)	−0.134 (−1.205–−0.064)	−0.175 (−0.257–−0.092)	−0.140 (−0.225–−0.056)
Professional title	−0.096 (−0.151–−0.040)		−0.186 (−0.265–−0.108)		−0.104 (−0.191–−0.017)	
Daily working hours	−0.250 (−0.330–−0.170)	−0.230 (−0.311–−0.148)	−0.275 (−0.390–−0.161)	−0.210 (−0.318–−0.101)	−0.280 (−0.406–−0.154)	−0.210 (−0.334–−0.086)
Average number of residents served per month	−0.033 (−0.082–0.015)		0.077 (0.009–0.146)		0.010 (−0.066–0.085)	
Average number of patients referred per month	0.020 (−0.045–0.086)		−0.045 (−0.137–0.048)		0.019 (−0.120–0.082)	
Average number of door–to–door services per month	0.046 (−0.012–0.104)		0.078 (−0.005–0.160)		0.006 (−0.085–0.097)	
Number of training sessions per year	0.190 (0.126–0.254)	0.157 (0.089–0.225)	0.473 (0.389–0.558)	0.401 (0.310–0.492)	0.350 (0.250–0.450)	0.232 (0.127–0.337)
Proportion of performance salary	0.007 (−0.047–0.061)		0.056 (−0.020–0.132)		0.040 (−0.045–0.124)	
	**Working conditions and environment**		**Interpersonal relationship**		**Management system**	
	**B(95%CI)**	AB^*^(95%CI)	**B(95%CI)**	AB^*^(95%CI)	**B(95%CI)**	AB^*^(95%CI)
Gender	0.314 (0.171–0.456)	0.264 (0.123–0.405)	0.255 (0.127–0.383)	0.258 (0.129–0.386)	0.307 (0.162–0.452)	0.206 (0.062–0.350)
Age	−0.134 (−0.231–0.037)		−0.071 (−0.159–0.016)		−0.144 (−0.243–−0.045)	
Income level	0.011 (−0.077–0.098)	0.105 (0.012–0.197)	−0.007 (−0.085–0.072)		0.057 (−0.032–0.146)	0.171 (0.077–0.265)
Education	−0.204 (−0.289–−0.119)		−0.117 (−0.194–−0.040)		−0.175 (−0.261–−0.088)	
Working years	−0.164 (−0.246–0.082)		−0.104 (−0.178–−0.031)		−0.224 (−0.307–−0.142)	−0.155 (−0.254–−0.056)
Professional title	−0.175 (−0.260–−0.089)	−0.203 (−0.297–−0.109)	−0.092 (−0.170–0.015)	−0.093 (−0.170–−0.015)	−0.159 (−0.246–−0.072)	−0.112 (−0.219–−0.005)
Daily working hours	−0.090 (−0.217–0.038)		−0.115 (−0.229–0.002)		−0.202 (−0.332–−0.073)	−0.191 (−0.322–−0.059)
Average number of residents served per month	0.033 (−0.042–0.108)		0.019 (−0.048–0.086)		0.064 (−0.012–0.140)	
Average number of patients referred per month	−0.006 (−0.107–0.095)		−0.020 (−0.110–0.069)		−0.063 (−0.166–0.039)	
Average number of door–to–door services per month	0.109 (0.020–0.199)		0.119 (0.039–0.199)		0.111 (0.019–0.202)	
Number of training sessions per year	0.332 (0.233–0.432)	0.260 (0.158–0.362)	0.180 (0.089–0.272)	0.124 (0.031–0.217)	0.333 (0.232–0.435)	0.247 (0.143–0.350)
Proportion of performance salary	0.087 (0.003–0.171)	0.096 (0.014–0.177)	0.079 (0.004–0.154)	0.091 (0.016–0.165)	0.081 (−0.005–0.167)	0.103 (0.018–0.187)
	**Social achievement**				**Cumulative satisfaction**		
		**B(95%CI)**	AB^*^(95%CI)		**B(95%CI)**	AB^*^(95%CI)
Gender		0.179 (0.056–0.303)	0.161 (0.038–0.285)		0.251 (0.148–0.353)	0.182 (0.084–0.280)
Age		−0.043 (−0.127–0.042)			−0.102 (−0.172–−0.032)	
Income level		0.018 (−0.058–0.093)			0.018 (−0.045–0.082)	0.092 (0.029–0.156)	
Education		−0.149 (−0.222–−0.075)	−0.092 (−0.169–−0.015)		−0.162 (−0.224–−0.101)	−0.091 (−0.155–−0.027)
Working years		−0.112 (−0.183–−0.040)			−0.158 (−0.217–−0.099)	−0.116 (−0.175–−0.056)
Professional title		−0.083 (−0.157–−0.008)			−0.129 (−0.191–−0.067)	
Daily working hours		−0.189 (−0.298–−0.080)	−0.144 (−0.255–−0.034)		−0.214 (−0.304–−0.123)	−0.180 (−0.270–−0.091)
Average number of residents served per month		0.026 (−0.039–0.091)			0.028 (−0.026–0.083)	
Average number of patients referred per month		0.035 (−0.052–0.122)			−0.016 (−0.089–0.057)	
Average number of door–to–door services per month		0.062 (−0.016–0.140)			0.071 (0.006–0.136)	
Number of training sessions per year		0.239 (0.154–0.324)	0.183 (0.091–0.276)		0.307 (0.237–0.378)	0.219 (0.145–0.292)
Proportion of performance salary		−0.007 (−0.078–0.065)			0.046 (−0.014–0.107)	0.058 (0.001–0.116)

Abbreviations: B, crude partial regression coefficient; AB, adjusted partial regression coefficient (significant predictors were included in the models using the stepwise model building strategy); 95% CI, 95% confidence intervals.

* Adjusted for the effect of region (Jinan, Weifang, Heze).

## Discussion

Since the implementation of the family physician contract system in 2016, China has established a family physician system that conforms to local characteristics through practical exploration in various regions. This has played a positive role in the rational utilization of health resources, reduction of medical and health costs, and improvement of residents' health conditions. However, the implementation of contracted services also increases the daily workload of the FPTMs. In the case of a small number of staff and a large number of work tasks, whether this will affect the work enthusiasm of family physician teams is an urgent issue that needs to be studied. This study conducted a survey on the job satisfaction of FPTMs and divided it into seven dimensions, which is conducive to finding the link of work enthusiasm, and provides a theoretical basis for the government and primary medical and health institutions to carry out targeted incentive measures.

Overall, the satisfaction of FPTMs was not high, which is consistent with the results of a study conducted in a city in eastern China (3.60 ± 0.70) (24). Satisfaction in the interpersonal relationship dimension was the highest. This shows that in the three relationships with superiors and subordinates, colleagues, and patients, FPTMs generally get along well with others, which is consistent with earlier research results (25). Satisfaction in the workload dimension was the lowest. With the change in direction of the “strengthening the grassroots level” of China's medical reform, various grassroots level policies have been continuously promoted. The family physician signing system is a new policy. As a result of the implementation of the family physician signing service, the workload of medical staff in the signing team has increased. Furthermore, the work content has changed, whereby medical staff are required to not only to provide basic medical services but also to provide basic public health services, and to not only passively sit down and complete basic medical services, but also to complete active door-to-door services (20, 26). The results of this survey show that the average working hours of medical staff is 8.65 h per day, exceeding the statutory 8-h working hours per day. Therefore, after the implementation of the new contracted services, local health administrative departments must also consider the relative shortage of personnel caused by the increase in workload. Moreover, there is a great lack of general practitioners, public health personnel, and nurses at the grassroots level (27). Therefore, we must adopt a more vigorous policy to attract medical personnel to the grassroots level for medical treatment and continue to strengthen the policy of order-oriented free medical students at the grassroots level, which has previously been widely implemented. In addition, we should also focus on strengthening medical education in general medicine, public health, and other majors in order to cultivate personnel that can join the family physician team in the future from a long-term perspective (28).

There are differences in the level of satisfaction between different regions, which follows the opposite trend to the level of economic development. This is not consistent with the conclusion that general practitioner satisfaction is higher in countries with higher GDP per capita based on the study of 34 countries (13). But this may be more in line with China's reality. Overall, in areas with low economic development, the cost level is not high, the pace of life is not fast, the pressure of life is small, and the expectation of all aspects of work is relatively low (21). At the same time, the implementation of the new policy brings financial subsidies for performance related pay. Therefore, the medical staff will be more motivated than before, and it will be easier to produce a sense of satisfaction, so the level of satisfaction is relatively high. However, this does not mean that this area does not need to continue to strengthen the measures to motivate the family physician team, and there is still a certain gap from the high level of satisfaction. Among the three regions, Jinan has the highest level of economic development, potentially because it is the provincial capital city with fierce job competition, scarce social resources, high living pressure, and relatively high expectations of various incentive measures, so the degree of satisfaction is relatively low. In future work, at the provincial level, we should consider the differences between regions with different levels of economic development, investigate incentive measures suitable for each region, and improve the satisfaction level of medical personnel (29).

Regression analysis showed that the satisfaction level of FPTMs was affected by factors such as “number of training sessions per year,” “qualifications,” “working hours per day” and “education level.” The first regards the number of training sessions, which had a positive effect on cumulative satisfaction and the seven dimensions of satisfaction. This demonstrates that medical personnel are highly satisfied with training opportunities, the fairness of opportunities, and training costs, which is related to the importance of grassroots medical and health institutions for training since the new medical reform. This also reflects that grassroots medical personnel have the most obvious desire for self-improvement. Research has confirmed that training can effectively improve the medical technology of grassroots medical personnel, and enhance their satisfaction (24, 30). Therefore, we should continue to strengthen the training of medical personnel, establish an optimal family medicine education and training system, and strengthen the skills of family physicians through training (31). Additionally, we should pay attention to the impact of other factors on satisfaction. Long working hours are the key factor that generally leads to low satisfaction of general practitioners. On the one hand, we should fundamentally continue to encourage medical students who can become general practitioners to work at the grassroots level, increase the supplement of grassroots human resources, and ease the contradiction between more tasks and less human resources. On the other hand, we should continue to increase the basic wage level of grassroots medical personnel. Through various effective incentive measures, we should improve the performance wage income of grassroots medical personnel, and improve the overall income level, improve the enthusiasm of medical personnel who work long hours every day (10).

Despite these findings, our study has some limitations. First, although the internal consistency of the measurement tools was good, there may have been some reporting bias in the self-reported data regarding job satisfaction. Second, although the factors of different levels of economic development were considered in the selection of sample areas, the selection of a wider geographical area will give rise to more robust and externally valid research conclusions, and future research should; therefore, be extended to more provinces and sample areas. In addition, although the selection of samples in this study has been scientifically selected and calculated, the number of samples selected by the three cities is not consistent, which may lead to sample bias to a certain extent, thus affecting the validity of the results of this study. Third, this study only reflects the cross-sectional survey status of satisfaction. After the policy has been implemented for a longer time, in future longitudinal research, we should continue to pay attention to the changes in the satisfaction of FPTMs and determine the positive factors to improve satisfaction.

## Conclusion

Family physicians play a key role in responding to the growing demand for primary healthcare due to aging. Conducting a survey with FPTMs, this study analyzed the current job satisfaction of FPTMs and its influencing factors after the implementation of the contract signing system in China. (1) The cumulative satisfaction of FPTMs is not high, so while developing contracted services for family physicians, China should also pay close attention to the workload and attitude of the medical staff, and adopt multiple approaches to increase the allocation of healthcare personnel at the community level and attract more general practitioners to work at the community level. (2) There are regional differences in FPTMs' satisfaction, which are influenced by many factors. Therefore, it is necessary to raise the salaries of primary healthcare workers, improve their material conditions, and pay attention to their self-improvement needs, which will contribute to increasing family physicians' satisfaction. Different incentive measures and strategies should be adopted in different regions to improve the overall work efficiency and service quality of FPTMs and the level of primary medical and health services.

## Data availability statement

The raw data supporting the conclusions of this article will be made available by the authors, without undue reservation.

## Ethics statement

Ethical review and approval was not required for the study on human participants in accordance with the Local Legislation and Institutional Requirements. Written informed consent from the participants was not required to participate in this study in accordance with the National Legislation and the Institutional Requirements.

## Author contributions

WC and YD wrote the preparation, visualization, and data management of the original draft, and organized the formation of the final manuscript. CW conceived the idea and designed the questionnaire. QJ organized the survey and data collection. RG, QG, FH, and WL participated in the fieldwork, discussion, and revision of the manuscript. All authors read and approved the final manuscript.

## Funding

This study was funded by the Youth Program of the National Natural Science Foundation of China (grant numbers 72104186 and 72004165), the Humanities and Social Science Research Youth Fund program of the Ministry of Education (grant number 20YJCZH002), the Natural Science Foundation Program of Shandong Province (grant number ZR2021MG019), and the Shandong Higher Medical Education Research Center Planning Program (grant number YJKT202126).

## Conflict of interest

The authors declare that the research was conducted in the absence of any commercial or financial relationships that could be construed as a potential conflict of interest.

## Publisher's note

All claims expressed in this article are solely those of the authors and do not necessarily represent those of their affiliated organizations, or those of the publisher, the editors and the reviewers. Any product that may be evaluated in this article, or claim that may be made by its manufacturer, is not guaranteed or endorsed by the publisher.
